# Release notice - Canadian Cancer Statistics 2023

**DOI:** 10.24095/hpcdp.44.1.04

**Published:** 2024-01

Just released!

Canadian Cancer Statistics 2023was released on 8 November 2023.

The publication of the Canadian Cancer Statistics 2023 was produced through a collaboration between the Canadian Cancer Society, Statistics Canada and the Public Health Agency of Canada, with data provided by the provincial and territorial cancer registries and analyses completed by Statistics Canada and the Public Health Agency of Canada. Canadian Cancer Statistics 2023 provides estimates of cancer incidence, mortality and survival for 2023.

**Highlights**

- For both males and females, lung cancer mortality has decreased by 3.8% per year since 2015. This represents the largest annual decline in mortality rates across all cancer types reported, and the fastest decline in lung cancer mortality reported to date in Canada.
- Probability of developing cancer over a lifetime is 45% and similar for males and females.
- For males, the largest decreases in cancer incidence were for: colorectal (−4.0% per year since 2014), lung (−2.6% per year since 2012) and leukemia (−2.0% per year since 2011).
- For females, the largest decreases in cancer incidence were for: colorectal (−3.1% per year since 2014), thyroid (−2.6% per year since 2012) and ovarian (−2.6% per year since 2014).
- The largest significant increase in cancer incidence among males was for melanoma (2.2% per year since 1984).
- Among females, cervical cancer is now the most rapidly increasing cancer (3.7% per year since 2015), marking the first significant increase in cervical cancer incidence since 1984.
- Approximately 1 in 4 Canadians are expected to die from cancer.
- The probability of dying from cancer is slightly higher for males (24%) than for females (21%).
- For males, the largest decreases in cancer mortality after lung (−4.3% per year since 2014) were for: bladder (−3.4% per year since 2016), kidney and renal pelvis (−3.1% per year since 2014) and melanoma (−2.6% per year since 2013).
- For females, the largest decreases in mortality after lung (−4.1% per year since 2016) were for: Hodgkin lymphoma (−3.2% per year since 1984), colorectal (−3.1% per year since 2014) and melanoma (−3.0% per year since 2014).

Access or download the latest Canadian Cancer Statistics and related resources.

